# Practical Food of Real Value

**Published:** 1917-11-03

**Authors:** 


### PRACTICAL FOOD OF REAL VALUE.

### I. WHY AND WHERE HOSPITALS COST MORE.

**By the House Governor of the London Hospital.**

WE all have some idea that it is very difficult to "carry on" in a voluntary hospital in war-time. It may be of some interest to the friends of the hospital outside, as well as to those who work inside, to know why. I suppose every housewife has discovered the enormously increased cost of the ordinary common things of the house; the hospital has also had to meet these increases in, for instance, coal, bread, meat, milk, brushes, and hardware; but it has also had to face increases which do not come into the housewife's ken at all—drugs, dressings, building material, uniforms, etc.

### Some Curious Facts.

We have just been going through our usual six-monthly business of contracting for some of our requirements, and the prices quoted to us have shown such astounding increases in cost that I have been looking into the question of increased cost generally, and in all departments. Everywhere it is the same. I have collected a good many of these, and perhaps some instances may be interesting, and it certainly is of interest to try to discover why they should have occurred. Why, for instance, should certain articles that come from Spain be almost unobtainable, although Spain is at war with no one? Why should belladonna, which grows quite well in England, have so gone up that atropine, which is made from it, is worth considerably more than its weight in gold? Why is it so difficult to obtain safety-pins and sheets and blankets and soap and cod-liver oil, and milk?

### Eatables First.

Let us first consider some of the things that are eatable, and meat is an important one. We contract for this every six months, as we do for other things. For the six months before war was declared we spent about £3,000 in meat. We are now spending £9,000 in six months. Here is the comparative price of the joints used in the hospital for nurses, resident doctors, and patients :

### Why Food is so Expensive.

We can easily guess why all these are so expensive. It is all a question of shipping. Ships are wanted for carrying troops and ammunition and food for our Armies in France and Salonika and Mesopotamia—work that is not required in peace-time. Therefore there is a shortage of ships for bringing chilled beef from the Argentine and mutton from Australia. And home-bred and home-fed meat is dear because the food on which they are fattened is dear—grain and oil-cake—and that is because of the shipping shortage again. Pork is dear because pigs are dear, and pigs are dear because pig-meal is dear. And home-fed meat is also dear because of the fact that the wages of those who look after cattle are higher. If there are three men for two jobs, wages are low; all the three men offer their labour at a lower and lower rate for fear of being the man left out. But if there are three jobs for two men, wages are high, because each employer offers more and more for fear he should be the one who cannot get his job done. Nowadays there are always only two men for three jobs. The third man is at the Front, and so wages are high, and these higher wages have to be added to the price of the thing the man produces, whether a pin or a pig—it is the consumer who pays.

### High Prices for Fish Explained.

Fish, too, is dear because the crews of our fishing fleets are busy fishing for something more important than haddock and herring. Our splendid fleets of mine-sweepers are all manned by the North Sea fishermen; indeed His Majesty's mine-sweeper 4526 is almost sure to be a steam trawler. Therefore the fish caught is not equal to the fish required; there is not enough to go round, and those who get it are those who are willing to pay most. Cod, plaice, turbot, fresh haddock, etc., cost before the war 7d. per pound all round. But there were people who, as the fish got short, offered more than 7d., say 8d., as they were anxious to have it. Therefore we had to offer 9d. Then they offered a little more. Again we had to offer still more. A hospital *must* have fish whoever goes without. We are now paying 1s. a lb. for fresh fish. Smoked haddock has advanced in the same way from 4d. to 9d. per lb. This increased cost of fish makes a difference of £800 in our six months' contract.

### A Great Rise in the Price of Eggs.

For a hospital, eggs are such an important part of the dietary that any great increase in their cost must be serious. They used to be imported in enormous quantities from Denmark, France, Spain, Holland, Italy, Russia, and even Egypt. All that is now stopped—the shipping difficulty again. We must now rely on our own fowl-rearing, and therefore the question of labour and cost of corn for feeding chickens influences the price. Eggs in pre-war days used to cost on an average ¾d. each; now we are paying 3¾d. each. For the six months before the war they cost £560; I expect that for the next six months they will cost £2,600; they certainly will if we use as many.

### Bacon, Bread, and Milk.

**Bacon** has gone up from an average price of 8d. per lb. to 1s. 9d. per lb. **Bread** is at the moment a little cheaper than it has been, but is still about double pre-war price. We expect to spend about £1,800 in bread and flour in the next six months. In the six months before the war we spent £911. This is a shipping rise. **Milk.**—The increase in the cost of milk is almost a tragedy for hospitals. It used to cost 8d. per gallon, and now costs 2s. 1d. per gallon. Our next six months' consumption will cost us £7,687 instead of about £2,500.

All **Vegetables** are "up." This is a labour rise. There have been times when our contractors could not deliver greens at *any* price. The greens were in the fields and we could have them if we would fetch them, but they (the contractors) had no labour to gather them.

### PRACTICAL FOOD OF REAL VALUE—*(continued)*.

Potatoes are twice the old price, and onions three times. **Tea** is another hospital requisite that has "gone up." It used to be (buying it, as we did, almost by the ton) 1s. 2½d. per pound. Now it is 2s. 1d. Coffee too has risen and costs exactly half as much again as it did. **Homely things**, such as rice, tapioca, treacle, oatmeal, pickles, are, on an average, three times as costly as before. Sugar—when we get it—is about twice its old price.

### Coal and Coke.

Now about some of the other things familiar to us all. The coal used in the ward fires has gone up from 21s. 6d. to 33s. 3d. per ton, and the coke from 16s. 7d. to 33s. 4d. The coal used in our great boilers in the basement is still more important. It must be the very best procurable, the very quality and kind the Fleet wants in such enormous quantities. And because the Navy wants so much, those who wish very much to have a share of what is left must be willing to pay a high price for it. And therefore we must be careful not to waste it. But how can *we* waste the boiler coal? We do not stoke the boiler fires and the hospital has to be kept at a certain temperature, and that is no concern of ours. *We*, however, can waste it by using more hot water than we need. From these big boilers, pipes carrying hot water run to every bathroom in the hospital and to every hot-water tap. It is as if a bit of the boiler ran all over the hospital. If you draw off a hundred gallons of hot water at one tap, the whole system feels it, and at one point a hundred gallons of *cold* water runs into the system to make up the loss. That cold water running in lowers the temperature of the whole system, and this shows on the gauge in the boiler-house at once; then the stokers throw a hundredweight or more of coal into the furnace to bring back the system to its proper temperature. If the 100 gallons of hot water had not been used, the gauge would not have moved and the stoker would not have had to "feed" his furnace. He just has to keep up enough fire to keep the gauge steady.

### Hard Shifts in Boiler-house.

There are well-known easy and hard shifts in the boiler-house. The hard shift is, of course, when we are all having hot baths. A couple of tons may be shot into the furnace at bath-time. I think it would be a splendid thing if all the other people in the hospital would be content with a little less hot water in their baths. But I cannot see why *I* should bother about such a small economy. *My* nice deep hot bath really won't matter. I'm only one. The great and important thing is that the other few hundred people should all honestly try to cut it down. But yet somehow the hot water does not come down, and the coal does go up. Perhaps—I wonder—you were just like me, and are leaving it to other people. A crowd is a lot of "*me's*." Well, I really and honestly will in my own bath cut down the hot water to three-quarters (as a start) what I should have used if I had not been visited by this attack of "conscience."

*(To be continued.)*

